# Effects of 12-Week Exercise Program on Enzyme Activity of Serum Matrix Metalloproteinase-9 and Tissue Inhibitor of Metalloproteinase-1 in Female Patients with Postmenopausal Osteoporosis: A Randomized Control Study

**DOI:** 10.1155/2020/9758289

**Published:** 2020-01-30

**Authors:** Tamara Filipović, Kristina Gopčević, Sanja Dimitrijević, Marija Hrković, Ana Backović, Milica Lazović

**Affiliations:** ^1^Institute for Rehabilitation, School of Medicine, University of Belgrade, Belgrade, Serbia; ^2^Institute for Chemistry in Medicine, School of Medicine, University of Belgrade, Belgrade, Serbia; ^3^Special Hospital for Cerebral Palsy and Developmental Neurology, School of Medicine, University of Belgrade, Belgrade, Serbia; ^4^Turval Laboratory Srl, Udine, Italy

## Abstract

**Background:**

Osteoporosis is a disease characterized by decreased bone density and destruction of bone microarchitecture. Indicators for altered bone homeostasis are changes in the serum level of matrix metalloproteinases (MMPs) and their tissue inhibitors (TIMPs). The purpose of the current study was to evaluate the effect of a 12-week exercise program on enzyme activity of serum MMP-9 and TIMP-1 in postmenopausal osteoporotic patients. *Materials and methods*. Participants were randomized in two groups: exercise (EG) (*N* = 37) and control (CG) (*N* = 37) and control (CG) (

**Results:**

Significant differences between pretreatment and posttreatment enzyme activities of serum MMP-9 (*p*=0.009), TIMP-1 (*p*=0.009), TIMP-1 (*p*=0.009), TIMP-1 (

**Conclusion:**

Our results suggest that a 12-week exercise program has an influence on enzyme activity of serum MMP-9, revealing a possible role of MMPs in initiating training-specific adaptation. Although measurements of circulating MMP-9 and TIMP-1 allowed us to detect effects of exercise, as of today, they have no real role in the diagnosis of osteoporosis and/or follow-up of osteoporotic patient's response to treatment. MMP-9 might be used as an important prognostic marker for the evaluation of patient's response to exercise. Larger-randomized controlled studies need to be performed to expand this area of knowledge. This trial is registered with trial registration number: NCT03816449).

## 1. Introduction

Osteoporosis is a chronic, progressive, and systemic metabolic bone disease characterized by low bone mineral density and microarchitectural changes of bone tissue leading to an increased tendency to fractures [1, 2]. Because of its multifactorial dimension, identifying the factors that are involved in osteoclast and osteoclast differentiation is as important as their dynamic changes, depending on the use of appropriate therapy [1].

Matrix metalloproteinases (MMPs) are a family of zinc dependent, proteolytic enzymes, responsible for extracellular matrix (ECM) degradation and cleavage of its structural components such as collagen and gelatin, thus having a crucial role in the process of ECM remodeling [3]. This process is important not only in bone formation but also in bone resorption, happening under physiological as well as pathological conditions, the latest arising mainly from altered MMP expression and activity causing abnormalities of the ECM remodeling [3, 4]. MMP expression and activity are very complex mechanism, regulated at different levels, including gene transcription, translation, secretion of the inactive proenzyme and proenzyme activation and inactivation via signaling from cytokines, growth and endocrine factors, integrins, and diverse ECM proteins [5, 6]. One of the principle modes of MMPs' regulation is through specific inhibition by direct binding to ECM secreted, tissue inhibitors of metalloproteinases, TIMPs (21–29 kDa) [7]. The association between MMP-1, MMP-2, MMP-3, MMP-9, and MMP-13 with the pathogenesis of osteoporosis has been demonstrated in different studies [5, 8, 9]. These are essential proteolytic enzymes responsible for early bone resorption because they degrade the collagen layer of the bone surface before demineralization begins [5, 8]. The complexity of the treatment of osteoporosis is reflected in the application of pharmacological and nonpharmacological therapy. According to the recommendations of the National Osteoporosis Foundation (NOF) bisphosphonates are still gold standard [1]. Nonpharmacological preventive measures include a modification of daily changes in physical activity, such as increasing weight-bearing exercises and muscle-strengthening exercise [1, 2]. Those exercises have an impact on slowing or stopping the loss of bone mass, improving balance, and reducing the risk of fall and fractures [1, 10, 11]. Weight-bearing exercise and resistance training might have an influence on osteogenesis only if the mechanical stress of long bones is strong enough [12]. This mechanical stress causes endogenous changes that interfere with bone remodeling, while the mechanical signal is being converted in biochemical signals that regulate bone activity mechanism [1, 2, 10–12].

Even if we assume that antiresorptive medicaments, together with the regular exercise program, are able to protect bone tissue to a similar extent [7], their mechanisms of action are most probably different. They may act on both organic and inorganic components, on both osteoblasts and the osteoclasts, and in both quantitative (protection of the bone) and qualitative sense (adequate primary and secondary mineralization, the formation of a normal organic matrix with suitable collagen fibers, etc.). The closer mechanism of these protective possibilities has to be clarified.

The serum MMP-9 concentration was observed to be negatively correlated with BMD (bone mineral density) and is considered as a biochemical marker of bone resorption and remodeling [13, 14] and an important marker in the early diagnosis of osteoporosis [8].

In bone tissue MMP-9 high specific degrading activity for denatured collagens in the ECM is highly expressed in osteoclasts, with a potential role in implantation and bone-resorbing activity of osteoclasts [5, 6, 15]. TIMP-1 is expressed from osteoblasts and osteocytes, and the activity of MMP-9 is likely to be inhibited by TIMP-1 [5]. Besides numerous experiments, a pathophysiologic understanding of MMP regulation focused on their mechanism of activation or suppression to date still remains partially resolved.

There is limited data of MMP-9 and TIMP-1 response on exercise. To our knowledge, there is no published data on enzyme activity of serum MMP-9 and TIMP-1 in postmenopausal women with osteoporosis involved in a 12-week exercise program performed with moderate intensity. In accordance with the current knowledge about the role of MMP-9 in bone remodeling and osteoporosis [3–5, 7, 16], we assumed that the serum level of MMP-9 could serve as an important marker for early assessment of treatment response. During this study, the enzyme activity of serum MMP-9, TIMP-1, as well as MMP-9/TIMP-1 ratio were observed in patients with postmenopausal osteoporosis, before and after prescribing to a 12-week exercise program [1, 2, 10–12, 17]. Therefore the aim of the study was to evaluate the enzyme activity of serum MMP-9 and TIMP-1 at baseline and after the 12-week period of supervised exercise program. MMP-9 activation was characterized by substrate gel electrophoresis (gelatin zymography) [18–20], while TIMP-1 by enzyme-linked immunosorbent assay (ELISA).

This study of a moderately sized, carefully defined group with well-constructed analysis sought to validate the effectiveness of a specifically designed exercise program on postmenopausal osteoporotic patient rehabilitation based on a careful evaluation of enzyme activity of serum MMP-9 and TIMP-1 at baseline and after the 12-week period.

## 2. Materials and Methods

### 2.1. Patient Population and Treatment

**Figure 1:**
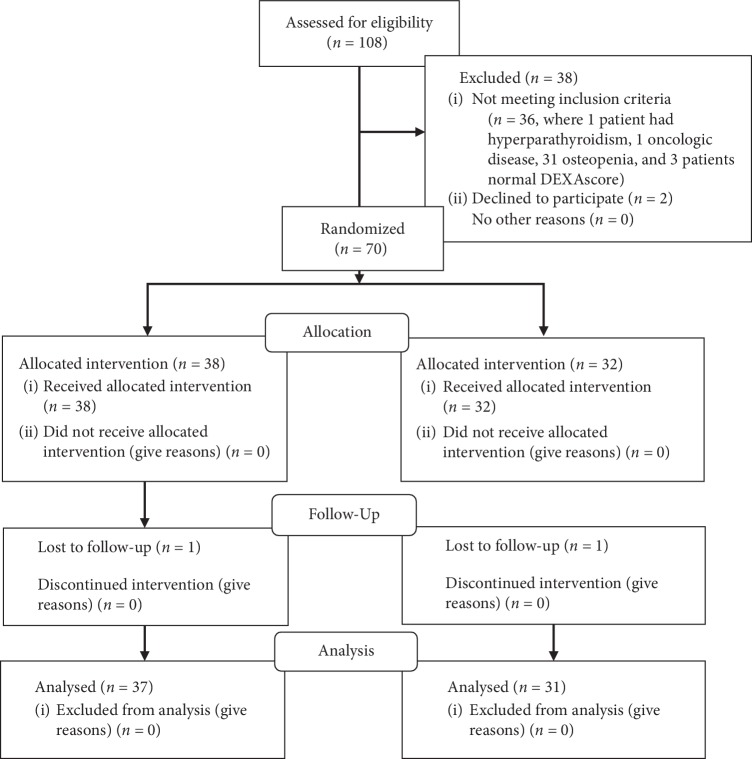
Participants flowchart.

Of the 108 patients who were screened for eligibility, 74 female patients were invited for participation. Of these, 72 potential participants were recruited for the study, of whom 70 showed up at the first visit and were randomized. The eligible consecutively recruited participants were randomly assigned in two groups in 1 : 1 ratio. In total, 37 patients in the exercise group (EG/aged 64.27 ± 5.61) and 31 in the control group (CG/aged 64.39 ± 4.50) accomplished the 3 months measurements and were included in the intent-to-treat analysis (Figure 1). Before starting the intervention, 70 female patients with postmenopausal osteoporosis started with regular medicamentous (bisphosphonate) therapy for osteoporosis according to the NOF recommendations [1]. Precisely, patients from both groups (EG and CG) have been treated with alendronate therapy and this treatment have not been changed during the study. Participants were highly motivated so there was high compliance (about 97%, 70 participants started the intervention, but two of them dropped off due to some personal reasons (one from the experimental, one from the control group); 68 of them completed the intervention period and were taken to final evaluation).

Standard recruiting criteria were as follows: (1) having postmenopausal osteoporosis diagnosed by central osteodensitometry (DEXA BMD, Osteosys: H2AY-002A, Seoul, Korea) according to T-score value at the lumbar spine and femoral neck (≤ −2, 5) [1, 2] at the Institute of rehabilitation, in Belgrade, (2) female patients of age between 65 and 70 years or even younger having any of the risk factors for osteoporosis (such as positive family history on a low-energy fracture, early menopause, prolonged amenorrhea) [1, 2], (3) being sedentary at the beginning of the study and during previous 6 months, which was conducted as not performing any exercise and fast walking for more than 15 minutes, more than twice/weekly for the above-mentioned period.

Exclusion criteria were as follows: previous history of osteoporotic fracture, perimenopausal status, metabolic bone disease, oncological diseases, hyperparathyroidism, corticosteroid therapy applied longer than 3 months, any hormonal therapy, liver and kidney dysfunction, resting blood pressure greater than 160/95 mmHg, and participation in any kind of physical activity for the last 6 months. The treatment consisted of a 12-week supervised exercise program proscribed to the patients in EG, while patients in CG did not take part in any physical activity for 12 weeks (during the study program).

All participants were informed about the study design. After getting written informed consent patients were physically examined and included in the study.

The study protocol was approved by the Ethics Committee of the Institute for rehabilitation, in Belgrade (Serbia), protocol number 02/2119-1, and reviewed and approved by ClinicalTrials.gov identifier (NCT number: NCT03816449). During the research, we took care to protect the privacy and anonymity of the respondent's data. The research was performed in compliance with the relevant laws and institutional guidelines.

### 2.2. Exercise Program

The exercise program designed by our research group has been previously described [21]. Shortly, the exercise group included 37 postmenopausal women who practiced the combined exercise program: aerobic exercise, resistance training, and balance exercise.

Rapid walking, 3–5 km/h, was prescribed as aerobic exercise. The intensity of the training was determined according to the maximal heart rate and was approximately 70% of its value [22]. Patients had this kind of activity five days per week, 50 minutes per day, for 12 weeks.

Resistance training and balance exercises were prescribed as a supervised group program. This program included balance exercises and exercises for strengthening muscles of the upper and lower extremities. The intensity of the “strength and resistance exercise program” was progressively increased on the weekly basis starting from 3 to 5 repetitions with no additional weight load per training going up to 8–12 repetitions with an extra weight load applied with the use of straps (“TheraBand,” Akron, Ohio-USA). Progression and maintenance of the weight load was decided together with the patient depending on the individual capacity [23]. The frequency of training was 3 times per week, each of 70-minute duration. The total exercise program lasted 12 weeks [21].

Certain functional assay [21] was used for monitoring the outcomes of the exercise performance. For the first 4 weeks patients had supervised exercise program with a trained exercise specialist at the Institute for Rehabilitation. After adopting technique of performing the exercises, participants continued to perform the same at home. They were told to write a diary of their regular exercise activity, while these dairies had been controlled three times during the study. Moreover, during the intervention period functional outcomes, such as Time-up and go test (TUG), sit to stand test (STS), and One leg Stance tests (OLST) had been conducted to evaluate patients' responses to exercise and objective the results. These tests had been observed before treatment and after the first and second months of treatment.

Patients from the control group (31 postmenopausal women) did not participate in any kind of exercise program, during the research period (12 weeks). For ethical reasons, after this period of time, the same exercise program had been proposed to patients from the control group, in order to accomplish complete treatment.

### 2.3. Testing Procedures

#### 2.3.1. Gelatin Zymography for the Assay of Enzyme Activity of Serum MMP-9

Pre- and poststudy blood samples were taken after 12 h overnight fast at 8.00-9.00 a.m. The blood was collected in vacutainers; the serum was separated by centrifugation (3000 rpm) and then stored at −20°C for future examination [18–20].

The activity of matrix metalloproteinase 9 (MMP-9) was determined by sodium dodecyl sulfate-polyacrylamide gel electrophoresis (SDS–PAGE) under nonreducing conditions and in gels with copolymerized gelatin [18–20]. MMP-9 (SIGMA-Aldrich, SAE0077 MMP-9-human, recombinant, ≥1,300 pmol/min/*μ*g, expressed in HEK 293 cells) was used as a standard for molecular weight calibration and was applied onto a gel without further preparation [19–25].

Briefly, appropriately diluted with 20% sucrose solutions, samples of sera from both groups, EG and CG, were mixed with buffer for treatment of samples contained: 0.125 M Tris-HCl; 4% SDS, 20% v/v glycerol; and 0.02% Bromophenol Blue, pH 6.8, in a volume ratio 1 : 1 and preincubated at 37°C for 45 minutes. Then, samples were applied onto wells (10 *μ*l/lane) and electrophoretically separated on 7.5% sodium dodecyl sulfate-polyacrylamide slab gels (Sodium Dodecyl Sulphate Polyacrylamide Gel Electrophoresis SDS–PAGE) containing copolymerized gelatin (1.0 mg/ml) under the conditions: *U* = 150 V, *A* = 50 mA, at +4°C for 90 minutes [18–20, 24].

Following electrophoresis, gels were washed three times for 20 min in renaturation buffer (2.5% Triton X-100), twice in double distilled water at +4°C, and then incubated for 24 h at 37°C in a buffer for enzyme assay (50 mmol/l Tris–Cl, pH 7.5 and 5 mmol/l CaCl_2_ in a volume 100 ml) in a thermostatically controlled water bath (Lab-ThermKühner Shaker, Kühner, Switzerland) at 50 rotations in a minute. After the incubation, gels were stained with 0.05% (w/v) Coomassie Brilliant Blue G-250 dye (CBB G-250) in a mixture of methanol: acetic acid: water (2.5 : 1 : 6.5) and destained with solution which contained 4% methanol (4%) and acetic acid (8%). The appearance of the clear zone to the dark blue background of the gel that was colored with CBB G-250 indicated the gelatinase activity [18–20, 24]. Finally, gelatinolytic activities were analyzed densitometrically using ImageJ 1.48v software (National Institutes of Health, Bethesda, MD, USA), calculating the densitometric value of the lyses against a dark blue background on zymography gels which quantified the surface and the intensity of the lysis bands after scanning of gels. MMP-9 electrophoretic migration was compared to known gelatinase molecular weight standard [20, 24]. Gels were photographed wet with background lighting and dried under vacuum between porous cellophane sheets and stored at room temperature. Gelatin substrate digestion levels were quantified as relative proteinase activity, comparing to relative activity of standard MMP-9, and recalculated according to activity of standard, which was applied in quantity of 10 ng/ml into corresponding concentrations of ng/ml.

#### 2.3.2. Enzyme-Linked Immunosorbent Assay (ELISA) for TIMP-1

TIMP-1 was determined using the enzyme-linked immunosorbent assay (ELISA) according to the manufacturer's recommendations.

The ELISA kit (Elabscience Biotechnology Inc., 2019–2020) was used for in vitro quantitative determination of human serum TIMP-1 with 0.10 ng/ml sensitivity, 0.16–10 ng/mL detection range, and no significant cross-reactivity or interference between Human TIMP-1 and analogues. Briefly, the Sandwich-ELISA principle used consists in the microplate precoated with a primary human anti-TIMP-1 specific antibody; TIMP-1 standards (for the standard curve formation) or study samples were added to the plate and let to combine with the specific antibody; then a secondary, biotinylated detection antibody specific for Human Ig G and Avidin-Horseradish Peroxidase (HRP) conjugate were added successively to each microplate well and incubated. Free components were washed away and finally the substrate solution was added to each well and left for the enzymatic reaction to occur. Only those wells that contained Human TIMP-1 appeared blue in color, which turned into yellow after the addition of stop solution and its optical density (OD) was measured at 450 nm ± 2 nm using a microplate reader (Rayto RT-6100). The OD values were then plotted on the standard curve for evaluation of TIMP-1 concentrations in each sample (sera of EG and CG).

### 2.4. Statistical Analysis

This is the primary analysis of these data. All statistical analyses were performed using the SPSS package program version 20.0 (IBM corporation). Complete-case analysis was performed without missing values imputation. Results were presented as mean ± standard deviation. Student's *t*-test or Mann–Whitney *U* test was used to determine the difference between the two groups. Differences between pre- and poststudy values of the intervention period were determined by using Wilcoxon Signed Ranks test.

The mean difference between the exercise and the control groups after 12 weeks was assessed by linear regression analysis (the mean difference is reported as *β*). Changes in MMP-9, TIMP-1, and MMP-9/TIMP-1 ratio were analyzed with the randomization group and baseline values as explanatory measures. Skewed continuous dependent variables were natural log-transformed before regression analysis. All *p* values < 0.05 (two-tailed) were considered significant.

## 3. Results

### 3.1. General Clinical Characteristics of Postmenopausal Osteoporotic Female Patients

Prestudy values of BMI (Body Mass Index), lumbar and neck *T*-score, biochemical parameters, and age were not significantly different between groups (EG and CG groups). Serum level of 25 (OH) vitamin D, total calcium, 24 h urinary calcium, Phosphate, 24 h urinary Phosphate, Creatinine, Creatinine Clearance, Albumin, Total Protein, Osteocalcin, Diuresis, Alkaline Phosphatase (ALP), Thyroid-Stimulating Hormone (TSH), Free Thyroxine (FT4), and Parathyroid Hormone (PTH) were measured at baseline in all participants. These parameters in both groups were insignificantly different (Table 1), except for Creatinine Clearance (*p*=0.017).

### 3.2. Changes during the Specific Exercise Program

**Figure 2:**
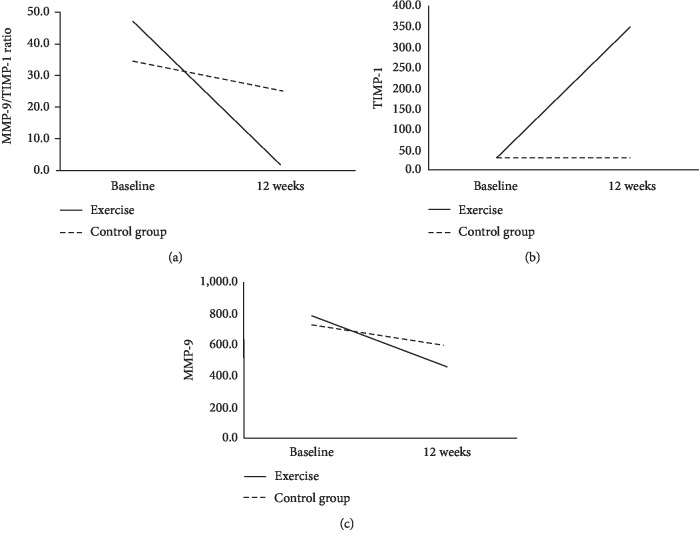
MMP-9, TIMP-1, and MMP-9/TIMP-1ratio followed over time in EG and CG.

Comparing the enzyme activity of serum MMP-9 (*p*=0.075) and TIMP-1 (*p*=0.777) at baseline, there was no statistically significant difference between groups. A significant difference was noted between pre- and postenzyme activity of serum MMP-9 (*p*=0.009), TIMP-1 (*p* < 0.001), and MMP-9/TIMP-1 ratio (*p* < 0.001) in EG (Table 2). No high significant differences have been noted between pre- and postenzyme activity of serum MMP-9 (*p*=0.583) and TIMP-1 (*p*=0.210) in CG (Table 2), only for the MMP-9/TIMP-1 ratio, a statistically significant change (*p*=0.028) was noted. The 12-week exercise program caused a significant decrease in the enzyme activity of serum MMP-9, as well as increased TIMP-1 in EG, while these differences have not been significant in CG (Figure 2).

Regression analysis demonstrated a significant mean difference in TIMP-1 after 12 weeks of follow-up between groups adjusted for age, baseline BMI, Vitamin D, and total PTH and Ca (*β* = −322.08 [95% CI-436.74–207.41]; *p* ≤ 0.001) and in MMP-9/TIMP-1 ratio after 12 weeks of follow-up between groups adjusted for age, baseline BMI, and Vitamin D (*β* = 24.02 [95% CI-13.32–34.73]; *p* ≤ 0.001). This result remained significant after adjustments for age, baseline BMI, Vitamin D, and total PTH and Ca: TIMP-1 (*β* = −318.32 [95% CI-433.44–203.21]; *p* ≤ 0.001), MMP-9/TIMP-1 ratio (*β* = 23.73 [95% CI-13.00–34.46]; *p* ≤ 0.001) (Table 3).

## 4. Discussion

It is well known that bone loss diseases, such as osteoporosis and rheumatoid arthritis, occur as a result of excessive bone resorption and bone remodeling imbalance correlated with increased catabolic processes and increased osteoclast activity [1, 2]. Enhanced osteoclast activity increases expression of MMP-9 which stimulates osteoclast reabsorption and degrades extracellular matrix proteins and collagen type I [5]. This role of MMP-9 is well documented in studies with wild-type mice which showed an excellent correlation between MMP-9 and invasion of osteoclasts into the core of diaphysis [13]. Moreover, studies on animal models also proved that MMP-9 can be a marker for osteoclast activity [5]. Widely used ovariectomized rat model recently showed a significant decrease in MMP9 activity, observed by means of gelatin zymography, after pharmacological treatment [14]. Finally, human studies confirmed the overexpression of MMP-9 in subjects suffering from osteoporosis [26].

Based on these facts, we hypothesized that well designed, controlled, 12-week exercise program could cause the inhibition of osteoclasts activity associated with the downregulation of MMP9 activity.

To test this hypothesis, we investigated changes in MMP-9 activity before and after the exercise program using gelatin zymography as a molecular technique.

In our study, we have tried to evaluate the response of enzyme activity of serum MMP-9 and TIMP-1 on appropriate treatment in postmenopausal osteoporosis, which must include pharmacological and nonpharmacological therapy. Taking into account the role of bisphosphonates in regulating activation pathways for MMPs in general and in osteoporosis [27, 28], as well as the necessity of proscribing adequate exercise program, we were interested in the role of supervised exercise program in this regulation, specifically. We proposed that pharmacological and nonpharmacological “agents,” working together, would have the ability to modulate MMPs activity in a period of 3 months. Studies on serum or plasma levels of gelatinase and their inhibitors showed an early release of MMP-9 after acute exercise of sufficient intensity, while data on TIMP-1 and the other MMPs were more contrasting. Most of the studies dealing with the effects of training indicated a trend toward reduction in blood gelatinase levels, once again more clear for MMP-9 which is in line with our results. The results were related to an anti-inflammatory effect of regular exercise and were more evident when training consisted of aerobic activities [7]. A few data available about resistance exercise suggest opposite effects on gelatinase concentrations [7, 29, 30].

**Figure 3:**
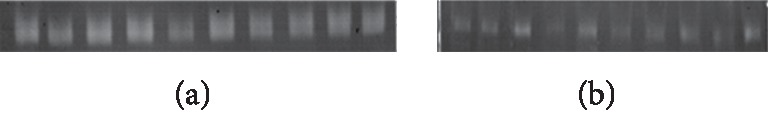
Serum activity of MMP-9 in the EG obtained by gelatin zymography. Representative protein bands of the MMP-9 protein are shown (a) before and (b) after the 12-week exercise program.

We reported decreased enzyme activity of MMP-9 (Figure 3), as well as increased TIMP-1 in the serum of female patients with postmenopausal osteoporosis, who had been involved in a 12-week exercise program, compared with those who have not got any physical activity treatment. These results point to the statistically significant reduction of the MMP-9/TIMP-1 ratio in EG (Figure 2).

On the contrary, in the CG, we reported no statistically significant slight increase of enzyme activity of serum MMP-9 and slight decrease of TIMP-1. These slight changes resulted in a statistically significant change in the MMP-9/TIMP-1 ratio in CG, which was probably induced by bisphosphonates therapy only. Comparing the results from both groups, we concluded that patients from the exercise group had better treatment.

In this study, we revealed a statistically significant decrease in the enzyme activity of serum MMP-9 in osteoporotic patients who had been training with resistance, walking, and balance exercise (Figure 3). The experimental evidence showed an early upregulation of MMP-9 and TIMP-1 expression by a specific 12-week exercise program. There is a growing body of evidence that MMP-9 and their tissue inhibitor do have important roles and show significant changes after exercise [29] which is in line with our results showing increased TIMP-1. Contrasting results have been published by Buyukyazi [31], who have reported no significant changes in the circulation of MMP-9 and TIMP-1 in postmenopausal healthy women after 8-week physical activity, which included walking only. Several studies demonstrated some acute changes in MMP-9 and TIMP-1 levels due to exercise [31]. These findings may suggest that there are some changes in the myofiber basement membrane via the MMP pathway in response to muscle damaging exercise [31]. The contradiction may result from the modes of the exercise programs since all the aforementioned studies measured the acute effects; however, we tried to demonstrate the chronic effects of endurance training in osteoporosis. Although a few papers reported decreased or unaltered circulating MMP-9 levels in osteoporotic patients on bisphosphonates therapy, none of them specially considered the influence of combined therapy (bisphosphonates and exercise) on the MMP-9 and TIMP-1 serum level. To our knowledge, this is the first study which underlined the tremendously important role of exercise in combination with bisphosphonate therapy and their influence in regulating the activity of serum MMP-9 and TIMP-1 in a follow-up period of 3 months in postmenopausal Serbian osteoporotic women. As we have shown in this paper, the possibility to assay the enzyme activity of serum MMP-9 and TIMP-1 may reflect a matrix turnover in osteoporotic patients according to the adequate treatment. Moreover, this can provide a valuable tool for evaluating osteoporotic patient response on well designed exercise training. Since MMP-9 was demonstrated to regulate the occurrence and development of osteoporosis [8] it may serve as a potential marker for the prediction and diagnostic method for postmenopausal osteoporosis. Also, if this marker was to be used clinically as a surrogate marker for techniques designed primarily or secondarily affecting ECM structure, consideration should be given to different reference ranges in younger and older osteoporotic patients, as well as their medical therapy. The facts described above suggest that the reduction of bone turnover is perhaps the most important factor during the course of osteoporosis, which can be achieved by using appropriate physical activity, as well as some pharmacotherapy. Thus the demonstration of MMP-9 and TIMP-1 in serum of female patients with postmenopausal osteoporosis provides information about bone turnover and its changing depending on exercise therapy.

## 5. Conclusion

The present findings indicate that exercise has significant effects on the enzyme activity of serum MMP-9 and TIMP-1 in humans, in vivo. Secondary, postmenopausal osteoporotic women can quickly reduce the risk of fall and improve their BMD, if they are willing to undergo intensive lifestyle modification. The real challenge is to maintain these lifestyle changes beyond 3 month exercise program. A moderate intensity, long duration program accompanied by pharmacotherapy and diet is proposed.

With its supervised prospective nature, this is the first study that examined changes in enzyme activity of serum MMP-9, TIMP-1, and MMP-9/TIMP-1 ratio in postmenopausal osteoporotic women following 12-week exercise program which included resistance training, balance exercise, and walking, besides medical therapy.

The main limitation of the study is its relatively small sample size and relatively small follow-up period, which may represent a source of bias. We had to form small groups due to the strict inclusion and exclusion criteria and the need for strict supervision.

Before our conclusions will be clinically applied, larger-randomized controlled multicenter studies will be required to validate our findings including the links (if any) to gene polymorphisms that may influence MMP/TIMP levels.

## Figures and Tables

**Table 1:** Initial clinical and biochemical characteristics for OP female patients in the exercise group and controls.

	Exercise *N* = 37	Controls *N* = 31	*p* value^1^
Age (years)	64.27 ± 5.61	64.39 ± 4.50	0.924
BMI	26.00 ± 4.48	25.42 ± 3.59	0.556
25 hydroxy vitamin D (nmol/L)	47.03 ± 17.51	45.61 ± 19.83	0.579^2^
Calcium, total (mmol/L)	2.44 ± 0.16	2.46 ± 0.21	0.659
24 hour urinary calcium (mmol/24H)	4.72 ± 4.07	4.61 ± 1.64	0.157^2^
Calcium ionized (mmol/L)	1.31 ± 0.26	1.34 ± 0.26	0.743
Phosphate (mmol/L)	1.120.21	1.34 ± 1.23	0.985^2^
24 hour urinary Phosphate (mmol/24H)	17.54 ± 7.79	14.32 ± 6.54	0.061^2^
Creatinine (*µ*mol/L)	67.39 ± 9.92	72.93 ± 7.69	0.128
Creatinine urine (mmol/24H)	8.96 ± 2.94	9.11 ± 2.10	0.392^2^
Creatinine clearance (ml/min)	87.06 ± 13.46	79.72 ± 11.14	0.017
Albumin (g/L)	48.41 ± 5.46	48.11 ± 4.54	0.804
Total protein (g/L)	75.56 ± 7.62	73.34 ± 5.80	0.178
PTH (ng/L)	55.00 ± 30.31	54.17 ± 20.89	0.618^2^
FT4 (pmol/L)	14.49 ± 2.98	13.95 ± 3.99	0.319^2^
TSH (*µ*IU/L)	2.62 ± 2.03	2.76 ± 1.16	0.162^2^
Osteocalcin (ng/ml)	24.14 ± 11.72	27.52 ± 9.43	0.048^2^
Diuresis (ml) Ƥ	2050.27 ± 472.80	2170.97 ± 466.15	0.209^2^
ALP (U/L)	72.59 ± 30.15	74.71 ± 32.23	0.554^2^
*T*-score *L*1–*L*4	−2.65 ± 0.89	−2.84 ± 0.74	0.268^2^
*T*-score neck	−2.59 ± 0.80	−2.42 ± 0.85	0.336^2^

^1^Data are expressed as mean ± SD. Biochemical data are before treatment. *p* < 0.005 vs. controls. ^1^Student's *t*-test. ^2^Mann–Whitney test.

**Table 2:** Changes in enzyme activity of serum MMP-9, TIMP-1, and MMP-9/TIMP-1 ratio in EG and CG.

	Baseline	12 weeks	*p* value^1^
MMP-9 (ng/mL)			
Exercise group	785.19 ± 511.76	456.57 ± 284.90	0.009
Control group	731.19 ± 788.3	595.06 ± 481.19	0.583
*p* value^2^	0.075	0.538	

TIMP-1 (ng/mL)			
Exercise group	27.59 ± 30.06	350.32 ± 313.93	<0.001
Control group	26.74 ± 28.75	32.64 ± 25.21	0.210
*p* value^2^	0.777	<0.001	

Ratio MMP-9/TIMP-1			
Exercise	46.97 ± 38.86	1.59 ± 0.82	<0.001
Control group	34.59 ± 40.69	25.37 ± 32.56	0.028
*p* value^2^	0.018	<0.001	

^1^Wilcoxon Signed Ranks test. ^2^Mann–Whitney test.

**Table 3:** The difference between enzyme activity of serum MMP-9, TIMP-1, and MMP-9/TIMP-1 ratio in the exercise and control groups after 12 weeks adjusted for baseline values of age, BMI, vitamin D, and total PTH and Ca.

	Adjusted^1^ *β*	95% CI	*p* value	Adjusted^2^ *β*	95% CI	*p* value
MMP-9 (ng/mL)	143.54	−45.13–332.21	0.133	147.08	−43.95–338.12	0.129
TIMP-1 (ng/mL)	−322.08	−436.74–207.41	<0.001	−318.32	−433.44–203.21	<0.001
Ratio MMP-9/TIMP-1	24.02	13.32–34.73	<0.001	23.73	13.00–34.46	<0.001

Adjusted^1^ *β*: adjusted for age, baseline BMI, and baseline Vitamin D. Adjusted^2^ *β*: adjusted for age, BMI, Vitamin D, total PTH, and Ca. 95% CI: 95% confidence interval.

## Data Availability

The data used to support the findings of this study are included within the article.
